# Supplementary material for: Absorption of Sulfur Dioxide by Tetraglyme–Sodium Salt Ionic Liquid
Source: Molecules. 2019 Jan 26;24(3):436. doi: 10.3390/molecules24030436 (PMC6384689; doi:10.3390/molecules24030436)
Supplement: Supplementary file 1 [file molecules-24-00436-s001.pdf]

Supporting Information

Study on Absorption of Sulfur Dioxide by Tetraglyme-Sodium salt Ionic Liquid

Qiang Xu[a], Wei Jiang[a], Jianbai Xiao[a], Xionghui Wei[a*]

[a] College of Chemistry and Molecular Engineering, Peking University, 100871, PR China

[*] Corresponding authors: Tel: +86-010-62751529. E-mail: xhwei@pku.edu.cn

Page No.	Contents
S-2	Experimental Section.
S-4	Figure S1 Mass spectrogram of [Na-tetraglyme][SCN] ionic liquid.
S-5	Figure S2 Thermal gravimetric analysis of tetraglyme and [Na-tetraglyme][SCN]. (At the constant temperature of 373 K)
S-6	Figure S3. SO_2 absorption capacities of tetraglyme and [Na-tetraglyme][SCN]at different SO_2 partial pressures. (SO_2 was diluted by N_2 or air)
S-7	Figure S4. SO_2 absorption capacities of [Na-tetraglyme][SCN] with different water contents at 293 K and 1 bar.

1. Experimental section

1.1 Chemicals

Tetraglyme (AR, 99%) was purchased from Shanghai Aladdin Bio-Chem Technology Co., LTD, and NaSCN, $NaBF_4$ and $NaClO_4$ were of analytical grade and purchased from Sinopharm Chemical Reagent Co.,Ltd. All reagents were used without further purification. Chromatographic grade ethanol and distilled water are also used for this work. Certified standard pure SO_2 gas (> 99.9%), N_2 (99.9% purity) and air (99.9%) supplied by Beijing Gas Centre, Peking University (China) is used to determine the SO_2 absorption capacity of ionic liquids.

1.2 Preparation of ionic liquids

The preparation of the ionic liquid was as follows: first, different sodium salts and tetraglyme were mixed at the stoichiometric ratio of 1:1, and then heated to a temperature of 303 K for 6 hours while maintaining sufficient agitation. The solution was then dried in a vacuum drying chamber for 48 hours. The resulting solution is transparent yellowish or colorless. According to Fig. S1 in supporting infomation, the cation of the ionic liquid formed at this time is a supramolecular system composed of sodium ions and neutral tetraglyme molecules. And the anion of the ionic liquid is still the anions initially introduced by the sodium salts. That is, the difference between these ionic liquids is mainly manifested in anions, and the cations have the same structure.

1.3 Absorption and desorption of SO_2

The absorption and desorption experiments of SO_2 were carried out in an absorption tube with an inner diameter of 15 mm and length of 150 mm. There is about 3 mL ILs added in the tube. Sulfur dioxide at a flow rate of 100 ml/min was bubbled through the absorbent sample containing absorber tube. The constant temperature required for absorption and regeneration is maintained by a circulating water bath into which the absorber tube is immersed. The absorption capacity of SO_2 was determined by means of weighing, and the analytical balance used was Sartorius BS 224S, which had an uncertainty of 0.1 mg. The absorption capacity has an uncertainty of 0.01 mol SO_2 / mol IL. In the absorption experiments under different pressures, a mixed gas having different partial pressures of sulfur dioxide was obtained by controlling the flow

rates of SO_2 and N_2. In the regeneration experiment, the temperature was maintained at 353 K, and the flow rate of nitrogen was 100 mL / min, and the analytical method used was similar to that of the absorption.

1.4 Spectral Measurements

MS, ^{1}H-NMR and ^{13}C-NMR and IR were all used to study the structure of the ionic liquids. ^{1}H-NMR, ^{13}C-NMR and IR were also used to study the nature of the interaction between ionic liquids and SO_2. The MS result came from Bruker Solarix XR and low resolution MS was applied to distinguish the cations. The instruments used in the ^{1}H-NMR and ^{13}C-NMR experiments were a 500 MHz Bruker Avance III spectrometer. NMR experiments were performed using an external standard method. Specifically, the sample was first injected into a custom glass tube with a size of 25 cm × 3 mm and the deuterated reagent was injected into an NMR tube with a size of 17.8 cm × 5 mm. Next, a capillary tube was inserted into the NMR tube to separate the sample from the deuteration reagent ($CDCl_3$). Infrared spectroscopy experiments were performed with a Bruker Vector 22 FT-IR spectrophotometer and analyzed with a typical thin film method. The instrument has a wavenumber range of 400 cm^{-1} to 4000 cm^{-1} with a resolution of 1 cm^{-1}.

Reference

1. C. Wang, Y. Guo, X. Zhu, G. Cui, H. Li, S. Dai, *Chem. Commun*. **2012**, *48*, 6526.

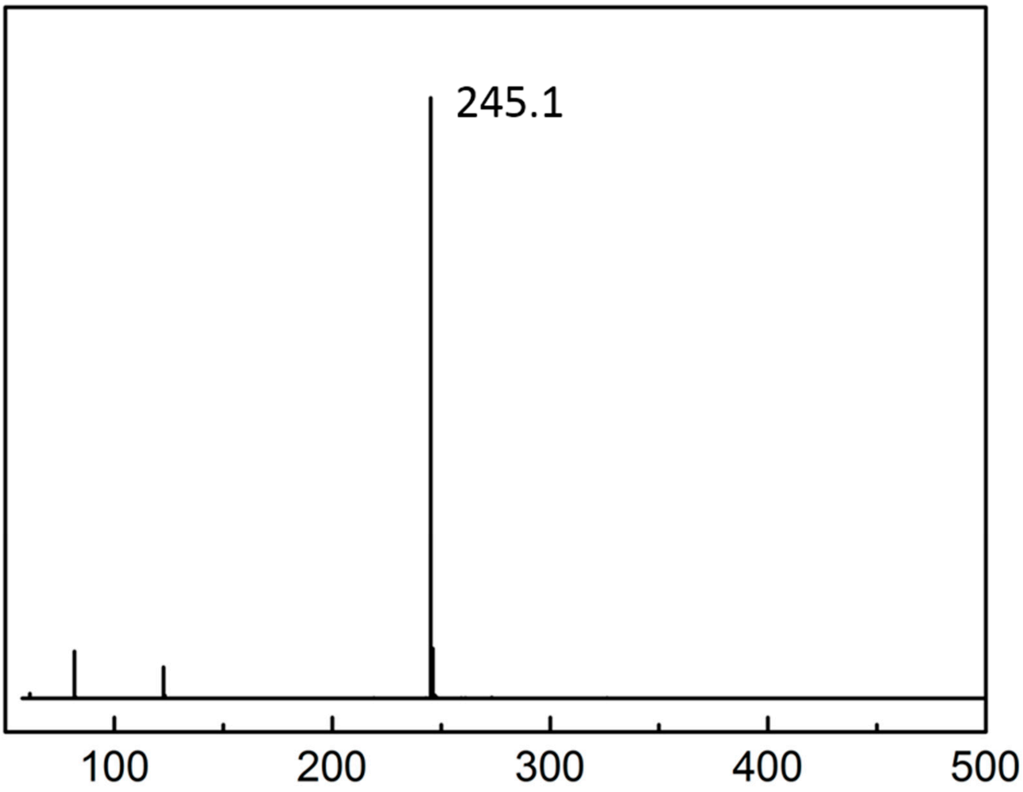

Figure S1. Mass spectrogram of [Na-tetraglyme][SCN] ionic liquid.

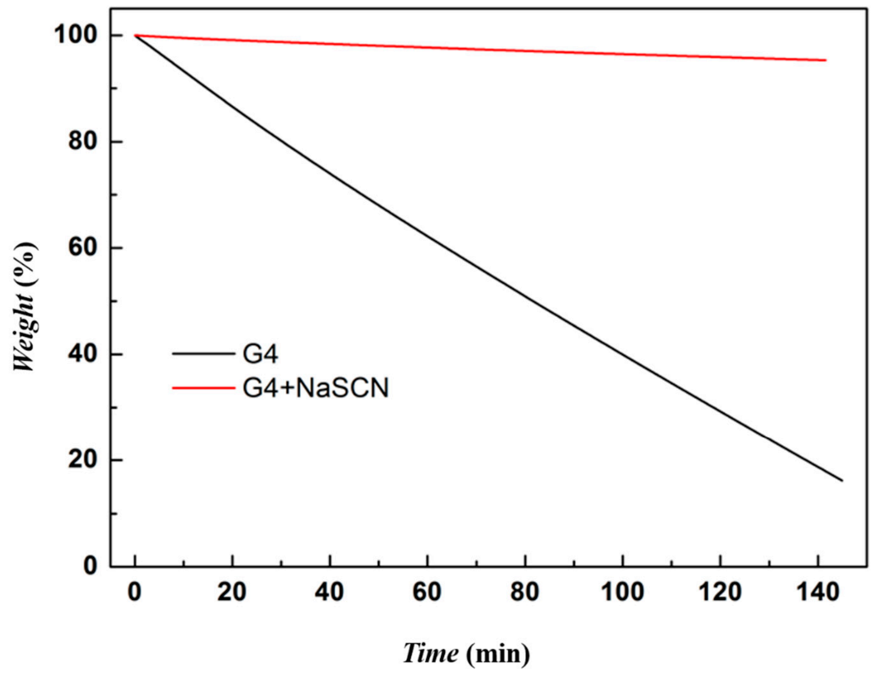

Figure S2. Thermal gravimetric analysis of tetraglyme and [Na-tetraglyme][SCN]. (At the constant temperature of 373 K)

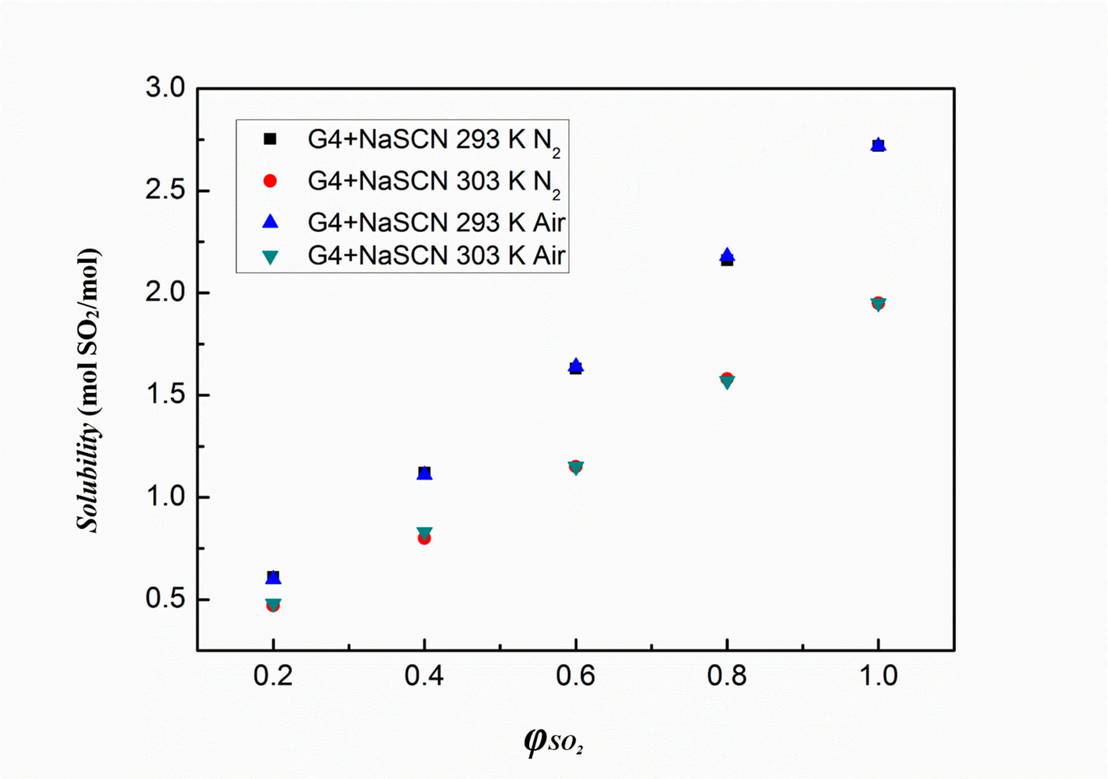

Figure S3. SO_2 absorption capacities of tetraglyme and [Na-tetraglyme][SCN]at different SO_2 partial pressures. (SO_2 was diluted by N_2 or air)

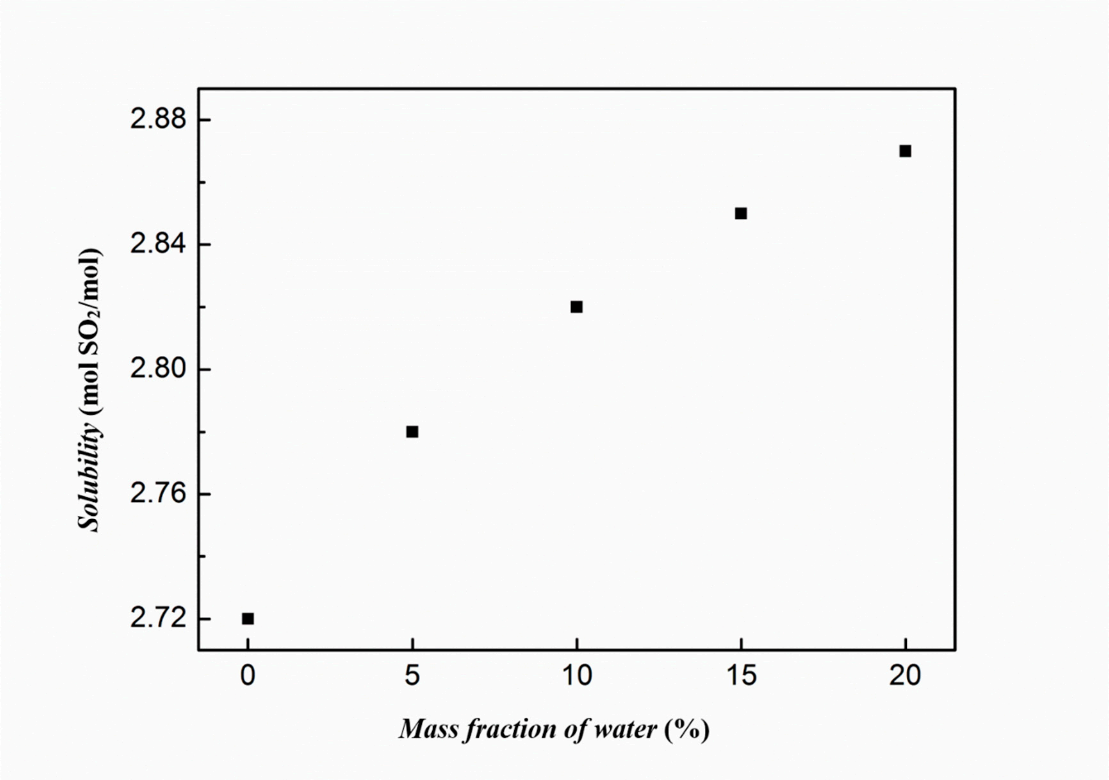

Figure S4. SO_2 absorption capacities of [Na-tetraglyme][SCN] with different water contents at 293 K and 1 bar.
